# Supplementary material for: LLAMA: a robust and scalable machine learning pipeline for analysis of large scale 4D microscopy data: analysis of cell ruffles and filopodia
Source: BMC Bioinformatics. 2021 Aug 19;22:410. doi: 10.1186/s12859-021-04324-z (PMC8375126; doi:10.1186/s12859-021-04324-z)
Supplement: Supplementary file 2 — Additional file 2. Supplementary Video 1. Supplementary Videos 1 and 2 demonstrate the key features of the visualiser and its use as part of the platform. Video 1 covers features using raster (voxel based) imaging, including training data selection and viewing image segmentations. [file 12859_2021_4324_MOESM2_ESM.docx]

**Video 1,2: LLAMA visualiser**

Two selected examples of tent pole ruffling events in LPS treated macrophage cells, showing LLS imaging and corresponding segmentation probability map. Segmentation colours are as described in Fig. 2. Videos show direct capture from the LLAMA visualiser with no additional image processing.

**Video 3,4: Tent pole ruffling**

Two selected examples of tent pole ruffling events in LPS treated macrophage cells, showing LLS imaging and corresponding segmentation probability map. Segmentation colours are as described in Fig. 2. Videos show direct capture from the LLAMA visualiser with no additional image processing.

**Videos 5-7:**

Representative examples selected from ten randomly sample ruffling events that include a “tent pole ruffle” configuration in which a pair of filopodia / tent poles are connected by a prominent ruffle. Segmentation colours are as described in Fig. 2. Videos show direct capture from the LLAMA visualiser with no additional image processing.
